# FMRI Approaches to Mapping Cerebrovascular Reactivity: Comparison of Gradient Echo BOLD and Spin Echo BOLD With Arterial Spin Labeling

**DOI:** 10.1002/mrm.70305

**Published:** 2026-02-22

**Authors:** Sara Pomante, Davide Di Censo, Alessandra Caporale, Fabrizio Fasano, Domenico Zacà, Elizabeth Jane Fear, Francesca Graziano, Manuela Carriero, Lucie Renee Raymonde Chalet, Emma Biondetti, Maria Eugenia Caligiuri, Michael Germuska, Richard Geoffrey Wise, Antonio Maria Chiarelli

**Affiliations:** ^1^ Department of Neurosciences, Imaging and Clinical Sciences University G. d'Annunzio of Chieti‐Pescara Chieti Abruzzo Italy; ^2^ Institute for Advanced Biomedical Technologies (ITAB) University G. d'Annunzio of Chieti‐Pescara Chieti Abruzzo Italy; ^3^ Siemens Healthcare Camberley Surrey UK; ^4^ Siemens Healthineers Forchheim Bavaria Germany; ^5^ Scientific Collaborations and Strategic Partnerships, Siemens Healthcare S.r.l. Milan Lombardia Italy; ^6^ Department of Medical and Surgical Sciences University Magna Graecia of Catanzaro Catanzaro Calabria Italy; ^7^ Neuroscience Research Center University Magna Graecia of Catanzaro Catanzaro Calabria Italy; ^8^ Department of Radiology University of California Davis Medical Center Sacramento California USA

**Keywords:** arterial spin labeling (ASL), cerebrovascular reactivity (CVR), gradient‐echo blood oxygen level dependent (BOLD) fMRI, spin‐echo BOLD fMRI

## Abstract

**Purpose:**

To compare markers of cerebrovascular reactivity (CVR) measured with Gradient Echo (GE, T2*w) and Spin‐Echo (SE, T2w) BOLD fMRI with quantitative physiological CVR measured using ASL.

**Methods:**

CVR, the ability of blood vessels to dilate and increase cerebral blood flow (CBF), can be investigated with ASL fMRI but has a low signal‐to‐noise ratio (SNR). GE‐BOLD provides semi‐quantitative CVR assessment, has high SNR but is influenced by baseline deoxyhemoglobin and the macrovascular venous compartment. SE‐BOLD is selectively more sensitive to microvasculature, mitigating contamination from larger vessels. We developed a pCASL GE‐BOLD/SE‐BOLD MRI sequence and collected all three fMRI modalities in 20 healthy subjects during a hypercapnic breath‐holding task.

**Results:**

CVRs in GM were CVR_ASL_ = 5.3%/mmHg ± 1.8%/mmHg (mean ± SD), CVR_GE‐BOLD_ = 0.18% BOLD/mmHg ± 0.05% BOLD/mmHg, and CVR_SE‐BOLD_ = 0.09% BOLD/mmHg ± 0.03% BOLD/mmHg. The ground truth of physiological CVR was represented by the ASL measurements with higher SNR (CVR_ASL_). CVR_GE‐BOLD_ and CVR_SE‐BOLD_ correlated across subjects with CVR_ASL_ in GM, with SE showing a stronger correlation (CVR_BOLD_ vs. CVR_ASL_:*r* = 0.54, *p* < 0.05 for GE‐BOLD and *r* = 0.70, *p* < 10^−3^ for SE‐BOLD). Region of interest (ROI) analysis based on previously reported macrovascular venous density maps derived from SWI showed that the lower correlation for GE‐BOLD was driven by GM ROIs with high venous density. Similar results were observed for spatial correlations (across regions) of group average maps of CVR (CVR_BOLD_ vs. CVR_ASL_:*r* = 0.44 for GE‐BOLD and *r* = 0.58 for SE‐BOLD, *p*'s < 10^−3^).

**Conclusions:**

BOLD fMRI provides a semi‐quantitative CVR assessment. Using SE‐BOLD may be advisable, as it approximates physiological CVR more closely than GE‐BOLD due to reduced sensitivity to larger veins while maintaining a similar group‐level sensitivity.

## 1 Introduction

Cerebrovascular reactivity (CVR) describes the ability of blood vessels to dilate, a necessary process to ensure that the blood supply to tissues meets demand. Vasodilation reduces cerebrovascular resistance thereby increasing cerebral blood flow (CBF) [1]. CVR can be evaluated by observing changes of perfusion in response to a vasodilatory stimulus [2]. These stimuli typically cause acidosis and a reduction in pH, which relaxes the smooth muscle cells in the walls of arteries and arterioles.

A common approach involves modifying the arterial CO_2_ partial pressure (PaCO_2_), either by inhaling CO_2_‐enriched gas or by voluntarily adjusting breathing patterns, most often through breath‐holding [3, 4]. Attempts to probe CVR by exploiting the endogenous modulations in the vascular tone induced, for example, by normal breathing, have also been reported [5, 6].

Different imaging techniques can map the hemodynamic changes triggered by the vasodilatory stimulus [7]. Positron emission tomography (PET), single‐photon emission computed tomography (SPECT) [8], and computed tomography (CT) [9] are among the methods used to measure CVR. However, these techniques involve exposure to ionizing radiation and generally offer low temporal resolution.

Magnetic resonance imaging (MRI) comprises various non‐invasive and non‐ionizing techniques that can measure the temporal evolution of brain hemodynamics and thereby probe CVR. Among the techniques that assess CBF are arterial spin labeling (ASL) [10] and phase contrast MRI (PC‐MRI) [11], in tissue and large vessels, respectively. However, these approaches either lack spatial specificity in the case of PC‐MRI or have a low signal‐to‐noise ratio in the case of ASL.

Due to its simplicity, broad availability and relatively high SNR, gradient‐echo (GE, T2*w) blood oxygen level dependent (BOLD) functional (f)MRI has been widely adopted as a marker of CVR in clinical research environments [7, 12, 13, 14, 15]. GE‐BOLD fMRI can track the decrease in the amount of the paramagnetic deoxyhemoglobin (dHb) in veins during hyperemia via its effect on transverse relaxation time (T2*). This has led to the widespread use of GE‐BOLD fMRI to study brain activity by relying on neurovascular coupling, where hyperemia follows an increase in neural activity. The same approach can be used to probe hyperemia during a vasodilatory stimulus which, unlike changing brain activity, is isometabolic, thus it is not associated with substantial changes in oxygen consumption, making the change in BOLD signal approximately proportional to the change in CBF [16].

However, the change in BOLD signal also depends on baseline dHb which can be expressed as a function of two physiological parameters: dHb concentration in blood ([dHb]), which itself can be expressed as a function of venous oxygen saturation (SvO_2_) and hemoglobin concentration in blood ([Hb]), and the volume that the deoxygenated blood occupies (CBV_dHb_), which is principally venous [17, 18, 19]. While hemoglobin concentration in blood and venous saturation in the brain exhibit low spatial variability, at least in healthy subjects, the voxel CBV_dHb_ may vary substantially, ranging from a few percent in the microvasculature to 100% in large veins [20].

The GE‐BOLD signal is known to be highly sensitive to large veins [21]. In fact, the GE‐BOLD signal response to a vasodilatory stimulus has been proposed as a marker of the spatial distribution of venous CBV [18]. To summarize, the GE‐BOLD signal, despite being sensitive to fractional changes in CBF, is also affected by venous oxygen saturation, hemoglobin concentration in blood, and CBV_dHb_, with the latter presenting large variabilities when considering both the micro and macrovascular compartments, and hence acting as a confounder on the estimate of the physiological CVR.

Spin‐Echo (SE, T2w) BOLD fMRI can be used as an alternative to GE‐BOLD fMRI [22]. SE‐BOLD fMRI exploits diffusion effects, which prevent the refocusing pulse from completely eliminating the extravascular effects of field distortions induced by dHb in small vessels that include capillaries and venules. The extravascular SE‐BOLD signal arises in the presence of rapidly spatially varying susceptibility‐induced magnetic field perturbations within the voxel, making it less sensitive to large venous vessels. As such, the SE‐BOLD is relatively more sensitive than GE‐BOLD to the microvascular compartment while being less sensitive to the macrovascular one [21, 23]. This effect, despite being associated with a reduction in SNR, may make SE‐BOLD fMRI better suited to probe physiological CVR than GE‐BOLD fMRI.

In this study, we compared markers of CVR based on GE‐BOLD and SE‐BOLD fMRI against the physiological CVR, based on CBF, measured using ASL. Unlike previous studies, which focused on comparing GE‐BOLD with ASL or comparing the two BOLD weightings [24, 25, 26, 27], often through asynchronous measures, we acquired all three weightings concurrently through a tailored GE‐BOLD, SE‐BOLD, and pseudo‐continuous ASL (pCASL) sequence. Vasodilation was induced through a breath‐holding task (breath‐hold, BH). To assess the impact of macrovasculature on BOLD CVR measures, we used atlas‐based reported values of macrovascular venous density derived from susceptibility weighted imaging (SWI) [28].

## 2 Methods

### 2.1 Participants

Twenty healthy subjects (7 females, 13 males; age: 34 ± 7 years) were recruited for the study, which was performed in accordance with the Declaration of Helsinki and approved by the Institutional review Board (RB) of the Department of Neurosciences, Imaging and Clinical Sciences, University “G. D'Annunzio” of Chieti‐Pescara, Italy. Written consent was obtained from each participant.

### 2.2 MRI Acquisition

Data were acquired on a 3T MAGNETOM Prisma scanner (Siemens Healthineers AG, Forchheim, Germany), equipped with a 32‐channel receive‐only head coil, located at the Institute for Advanced Biomedical Technologies, University “G. D'Annunzio” of Chieti‐Pescara, Italy.

**FIGURE 1:**
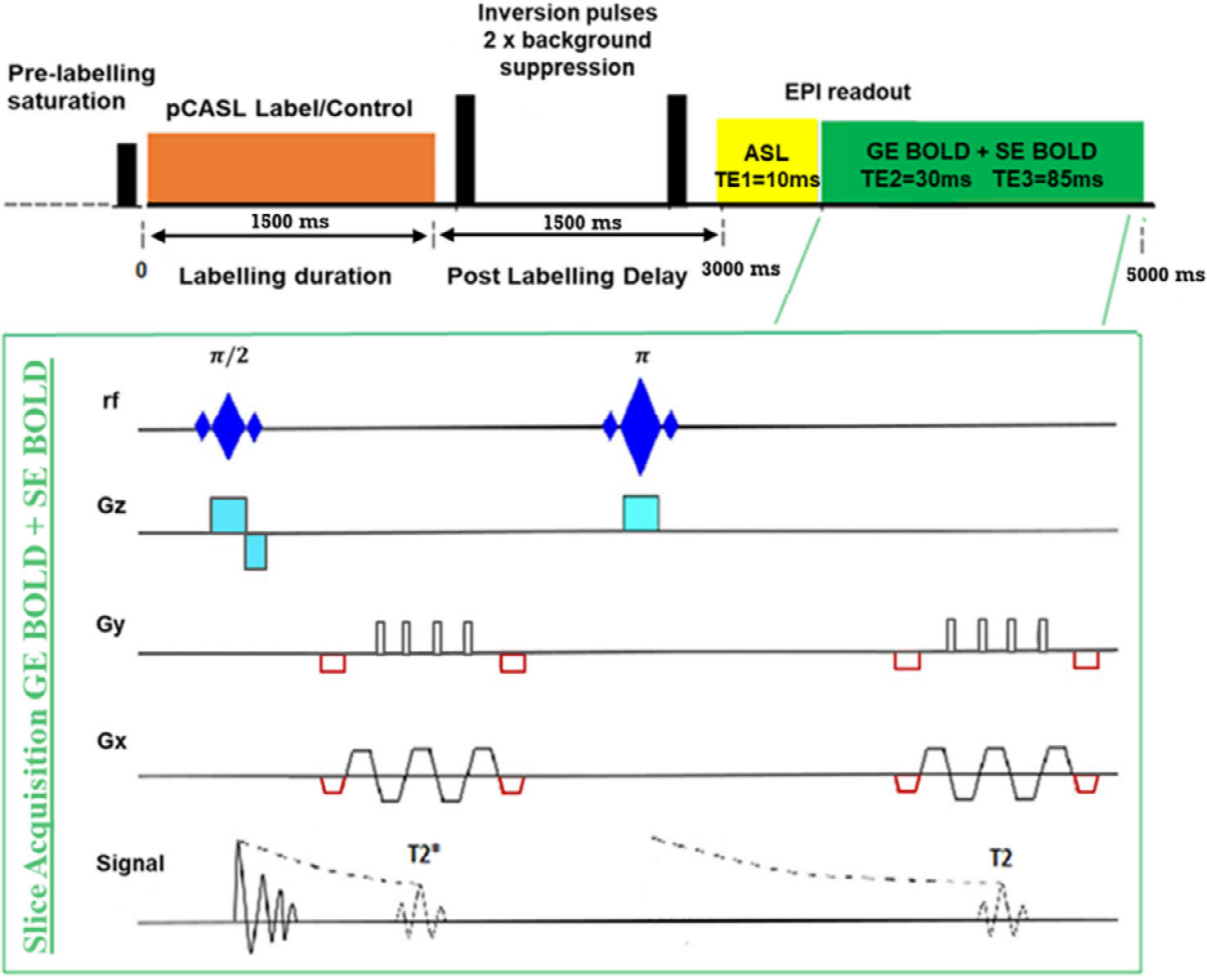
Gradient Echo (GE)/Spin Echo (SE) pseudo‐continuous arterial spin labeling (pCASL) sequence implemented for the study. The sequence allowed for the acquisition of the three fMRI weightings used to infer the different cerebrovascular reactivities. For each repetition time (TR), the sequence involved saturation within the field of view, pCASL labeling, two inversion pulses for background suppression in the field of view, and 2D EPI readouts separated into two excitation modules: one at the optimal suppression where the ASL slices were acquired, and another where the BOLD slices were acquired. Each BOLD slice acquisition involved excitation, EPI GE readout, refocusing, and EPI SE readout.

The fMRI recordings were performed using a tailored pseudo‐continuous (pC)ASL research sequence (Figure 1), based on the vendor's product. We developed an ASL‐BOLD sequence relying on a dual‐excitation (DEXI) echo planar imaging (EPI) 2D readout consisting of a short echo time, TE1 = 10 ms for ASL, and following a second excitation, a longer echo time, TE2 = 30 ms for GE‐BOLD [17, 29]. Additionally, a refocusing pulse was inserted after the second excitation for each slice to produce SE weighting with an echo time for optimal sensitivities to T2 weighted signal changes of TE3 = 85 ms. The labeling scheme of pCASL was balanced (flip angle: 20°; RF pulses: Gaussian, 600 μs duration, 1 ms separation) with gradient amplitudes in the range of optimal values to minimize velocity sensitivity (mean tagging gradient (*G*_avg_): 0.8 mT/m; tagging gradient during RF pulses (*G*_max_): 6 mT/m; *G*_max_/*G*_avg_ = 7.5) [30, 31]. Pre‐labeling saturation and two inversion pulses for background suppression were included [31]. The two separate readouts were used for optimal background suppression at the short TE and minimal suppression at the long TE. The labeling duration (τ) and the Post‐labeling delay (PLD) were both set to 1.5 s, GRAPPA acceleration was used (factor = 3). An effective TR of 5 s was employed to acquire 14 slices, with an in‐plane resolution of 3.4 × 3.4 mm^2^ and a slice thickness of 7 mm with a 30% slice gap to cover the entire brain.

fMRI data were acquired during a BH task. The BH protocol consisted of an initial baseline of 60 s and 10 cycles of post‐expiratory breath‐holds, each lasting 20 s, with a 40‐s normal breathing recovery interval, resulting in an acquisition duration of 11 min and 28 s [4]. Subjects were visually prompted with instructions projected onto the center of the screen against a gray background, which were implemented in E‐Prime 3.0 stimulus presentation software. They were instructed to maintain a neutral diaphragm position during BH and to fully breathe out to expel any residual air from the lungs at the end of each BH. This was to enable the estimation of the arterial content of CO_2_ at the end of the BH [32].

During the fMRI recordings, CO_2_ partial pressure in the expired air was measured via a nasal cannula connected through a sampling line to a gas analyzer system (ADInstruments, Dunedin, New Zealand). The continuous recording of CO_2_ partial pressure was performed using LabChart software (ADInstruments, Dunedin, New Zealand).

Two proton density images (M0) were acquired for susceptibility distortion correction of fMRI data and ASL calibration with pCASL labeling and background suppression pulses switched off, with TR = 7 s and TE = 10 ms, and opposite phase encoding directions (anterior–posterior and posterior–anterior, AP and PA).

A magnetization‐prepared rapid acquisition with two gradient echo (MP2RAGE) T1‐weighted scan was acquired for registration and brain segmentation purposes (matrix 144 × 198 × 224, 1.2 mm isotropic resolution, TR/TE = 4190/3.49 ms, TI1/TI2 = 700/2500 ms).

Table 1 summarizes the parameters of the main MRI sequences employed.

**TABLE 1:** MRI protocols and acquisition parameters.

Protocols/Parameters	3D T1‐weighted structural MRI	4D ASL and T2*‐weighted BOLD fMRI and T2‐weighted BOLD fMRI
Sequence	MP2RAGE	PCASL DEXI GE + SE EPI
Resolution (mm^3^)	1.2 × 1.2 × 1.2	3.4 × 3.4 × 7.0
Matrix size (voxels)	144 × 198 × 224	64 × 64 × 14
Slice gap (mm)	—	30%
Pixel bandwidth (Hz/Px)	180	2112
Echo time (TE) (ms)	3.49	TE1 = 10
	—	TE2 = 30
	—	TE3 = 85
Repetition time (TR) (ms)	4190	5000
Inversion time (TI) (ms)	TI1 = 700	—
	TI2 = 2500	—
Labeling duration (τ) (ms)	—	1500
Post labeling delay (PLD) (ms)	—	1500
Grappa acceleration	3	3

### 2.3 PetCO_2_ Calculation

End‐tidal CO_2_ partial pressure (PetCO_2_), taken as a surrogate measure of arterial CO_2_ partial pressure, was extracted using in‐house software in Matlab (MATLAB version: 9.13.0 (R2022b), Natick, Massachusetts: The MathWorks Inc.; 2022) [17, 33].

The PetCO_2_ trace was obtained by identifying and linearly interpolating the expiratory peaks of the raw CO_2_ trace. The raw PetCO_2_ signal was resampled at the fMRI TR, shifted to account for time lags between expiration and CO_2_ concentration recordings outside of the scanning room, and band‐pass filtered (zero‐phase forward and reverse filtering with digital IIR Butterworth digital filter, order 4) with cut‐off times of 10.1 s (considering a Nyquist time of 10 s) and 150 s (6.7–99 mHz).

### 2.4 fMRI Processing

The processing steps involved data normalization, motion correction, susceptibility distortion correction, filtering, and regression (please refer to Figure S1).

The MP2RAGE sequence at the two inversion times was used to extrapolate the MPRAGE T1 weighting image, which was then employed for warping into MNI space (antsRegistration, SyN, ANTs) and for tissue segmentation (FAST, FSL) [34, 35, 36].

The two M0s were corrected for susceptibility distortion (Topup, FSL) [37] and intensity inhomogeneity (N4biasfieldcorrection, ANTs) [38]. The corrected M0 image, skull‐stripped using FSL BET, was then rigidly registered to the T1‐weighted image, and the transformation matrix was inverted to bring the GM and white matter (WM) partial volume estimates into the M0 space, which were then thresholded (threshold = 0.5) to obtain compartmental masks.

GM region of interest (ROI) analysis was based on the Harvard‐Oxford probabilistic Atlas covering 48 bilateral cortical areas [39], for which estimates of average macrovascular venous density were available from previous studies [28]. The Atlas was warped into the M0 space from MNI.

The BH fMRI data were processed using FSL, ANTs, and in‐house algorithms in Matlab [34, 35]. An ad‐hoc fMRI motion correction pipeline was implemented to minimize misregistration effects introduced by the difference in contrast between ASL tag and control images [40]. The three BH fMRI time courses were split based on tag and control and registered to the respective first volumes using FLIRT from FSL [41, 42, 43]. Then, the first volume of the control images was registered to the first volume of the tag images using transformations computed only on GE‐BOLD weighting (which showed minimal perfusion weighting and had a higher SNR compared to SE‐BOLD, FLIRT from FSL). All other control volumes for each weighting were registered using the derived transformations.

The motion corrected volumes were then rigidly registered to the brain‐extracted M0, which was acquired with the same phase encoding direction as the functional scans (anterior–posterior), using ANTs with the transformation matrix computed only on the shortest TE weighting. The fMRI volumes for each echo were then corrected for susceptibility distortions using the FSL ApplyTopup function.

In‐house algorithms in Matlab were used for further analysis. Surround subtraction was performed on the ASL volumes to obtain a perfusion signal proportional to the CBF [44]. Surround averaging was performed on the BOLD signals to eliminate perfusion contamination. CBF and BOLD signals were expressed as relative changes with respect to the baseline (estimated by averaging the first 50 s of recordings). All three fMRI signals were band‐pass filtered (zero‐phase forward and reverse filtering with a digital IIR Butterworth digital filter, order 4) with cut‐off times of 10.1 s (considering a Nyquist time of 10 s) and 150 s (6.7–99 mHz).

The evaluation of the voxel‐wise BOLD and CBF CVRs, expressed as the signal percent change per unit of PetCO_2_ modulation, was implemented using linear regression [45], where the filtered PetCO_2_ trace was used as the independent variable. The resampled and filtered PetCO_2_ was allowed to shift by ±10 s to account for hemodynamic lags [4]. The time lag was evaluated independently for each fMRI weighting.

The regression β‐weights delivered an estimate of CVR_GE‐BOLD_, CVR_SE‐BOLD_, expressed as % BOLD/mmHg, and CVR_ASL_, expressed as %CBF/mmHg changes [46]. The confidence in the CVRs estimate was evaluated by dividing the GLM β‐weight by its confidence interval (*z*‐scoring).

To assess associations between the parameters of interest across subjects, global and ROI estimates of CVRs were performed. We consider CVR_ASL_ as the reference in this study as it represents a single physiological quantity. In order to improve confidence in the results, given the low SNR of ASL, CVRs estimates were evaluated also as a function of the confidence in the voxelwise CVR_ASL_ estimates (a mask was created by defining a lower bound threshold on the *z*‐score of the CVR_ASL_ considered in the analysis and applied to all three fMRI weightings). For the across‐space analysis and for the evaluation of average maps and their statistical significance across subjects, CVR_GE‐BOLD_, CVR_SE‐BOLD_, and CVR_ASL_ maps were warped into the 2‐mm isotropic MNI standard space with intermediate transformation to the structural T1‐weighted space, which was used to compute the non‐linear transformation to the template space.

### 2.5 Statistical Analysis

Pearson's or Spearman's correlations were performed to assess associations between the CVRs. *T*‐tests were performed to assess second‐level, across subjects, voxel‐wise statistical significance. To assess spatial correlations as a function of the number of subjects considered in the averaging, bootstrapping with 100 iterations for each averaging number was used [47]. A *p* < 0.05 was considered statistically significant. False discovery (FDR) was used to account for multiple comparisons.

## 3 Results

The average PetCO_2_ at rest was 35.0 ± 1.4 mmHg (mean ± SD). The BH task was successfully performed by all participants and induced a consistent modulation in PetCO_2_.

**FIGURE 2:**
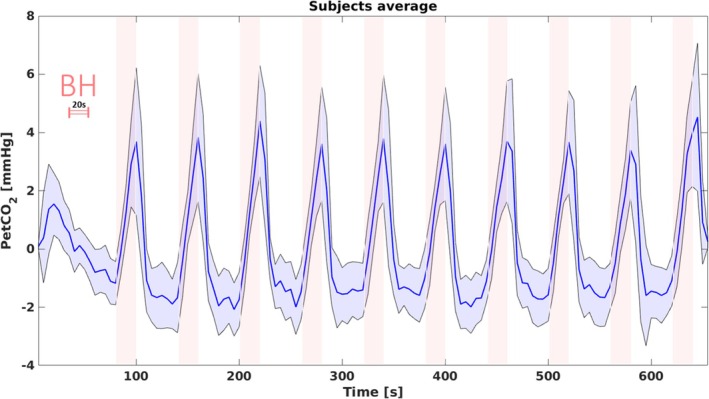
Average (and standard deviation) trace of the change in PetCO_2_ (filtered signal) across the study participants, showing the hypercapnic effect of the repeated breath‐holding stimulation.

Figure 2 shows the mean and standard deviation (SD) of the filtered PetCO_2_ traces across subjects during the BH protocol. The average modulation in PetCO_2_ (defined as the difference between the 95th and the 5th percentiles of the filtered PetCO_2_ signal) was 6.7 ± 2.1 mmHg.

**FIGURE 3:**
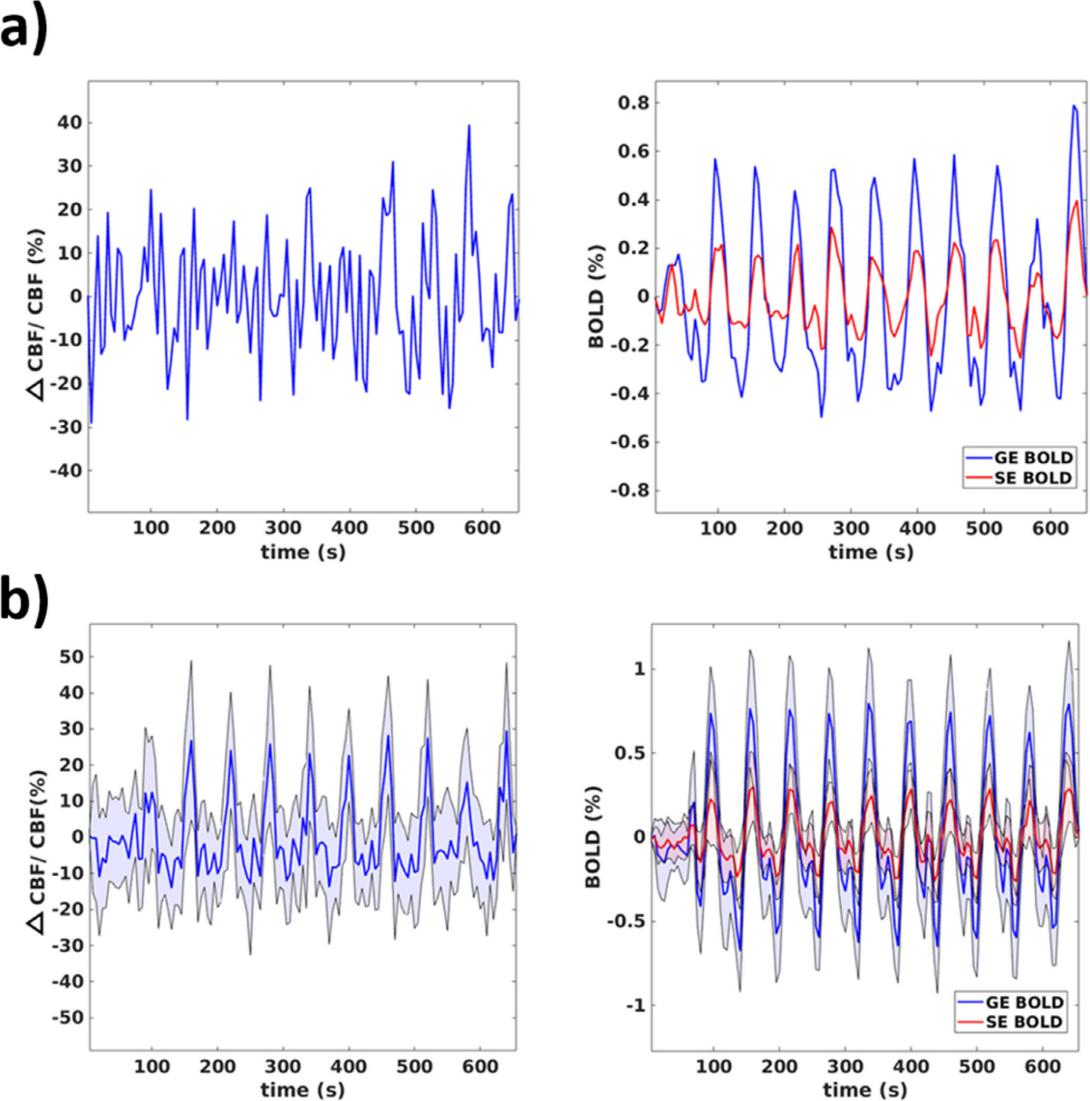
Examples of (a) filtered fractional change in cerebral blood flow (CBF) and filtered GE‐BOLD and SE‐BOLD signals during the breath‐holding task in one subject; (b) same as in (a) but averaged across subjects.

Figure 3 reports temporal CBF and BOLD signal modulations and retrieved CVR maps. Figure 3a shows the changes in CBF and BOLD signals for a single subject in GM, whereas Figure 3b reports the average values across subjects. The average GM CBF and BOLD signal changes (defined as the difference between the 95th and the 5th percentiles of the filtered signals) were 34% ± 12%, 1.48% ± 0.10%, 0.65% ± 0.22%, respectively for GM CBF, GM GE‐BOLD, and GM SE‐BOLD signals.

**FIGURE 4:**
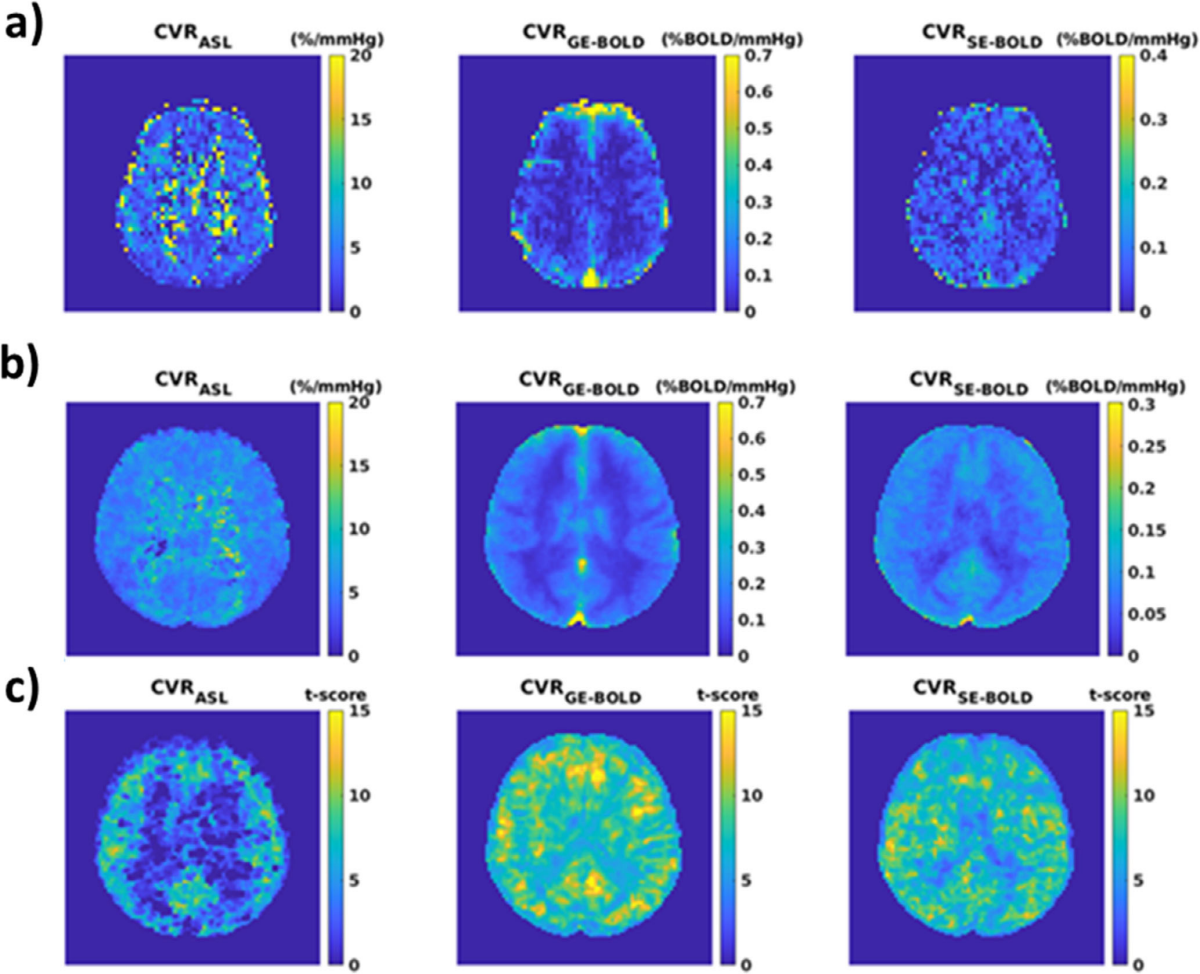
Example of (a) single‐subject CVR_ASL_, CVR_GE‐BOLD_ and CVR_SE‐BOLD_ maps; (b) subjects' average CVR_ASL_, CVR_GE‐BOLD_, and CVR_SE‐BOLD_ maps computed in the MNI space; (c) second‐level, across subjects *t*‐score maps for CVR_ASL_, CVR_GE‐BOLD_, and CVR_SE‐BOLD_ computed in the MNI space.

Figure 4a presents examples of calculated CVR maps in one subject, while Figure 4b,c report group average maps and *t*‐score maps across subjects computed in MNI space. While not reported, time‐lag maps were consistent across modalities, particularly in voxels with highly significant correlation between the PetCO_2_ trace and the fMRI signals.

**FIGURE 5:**
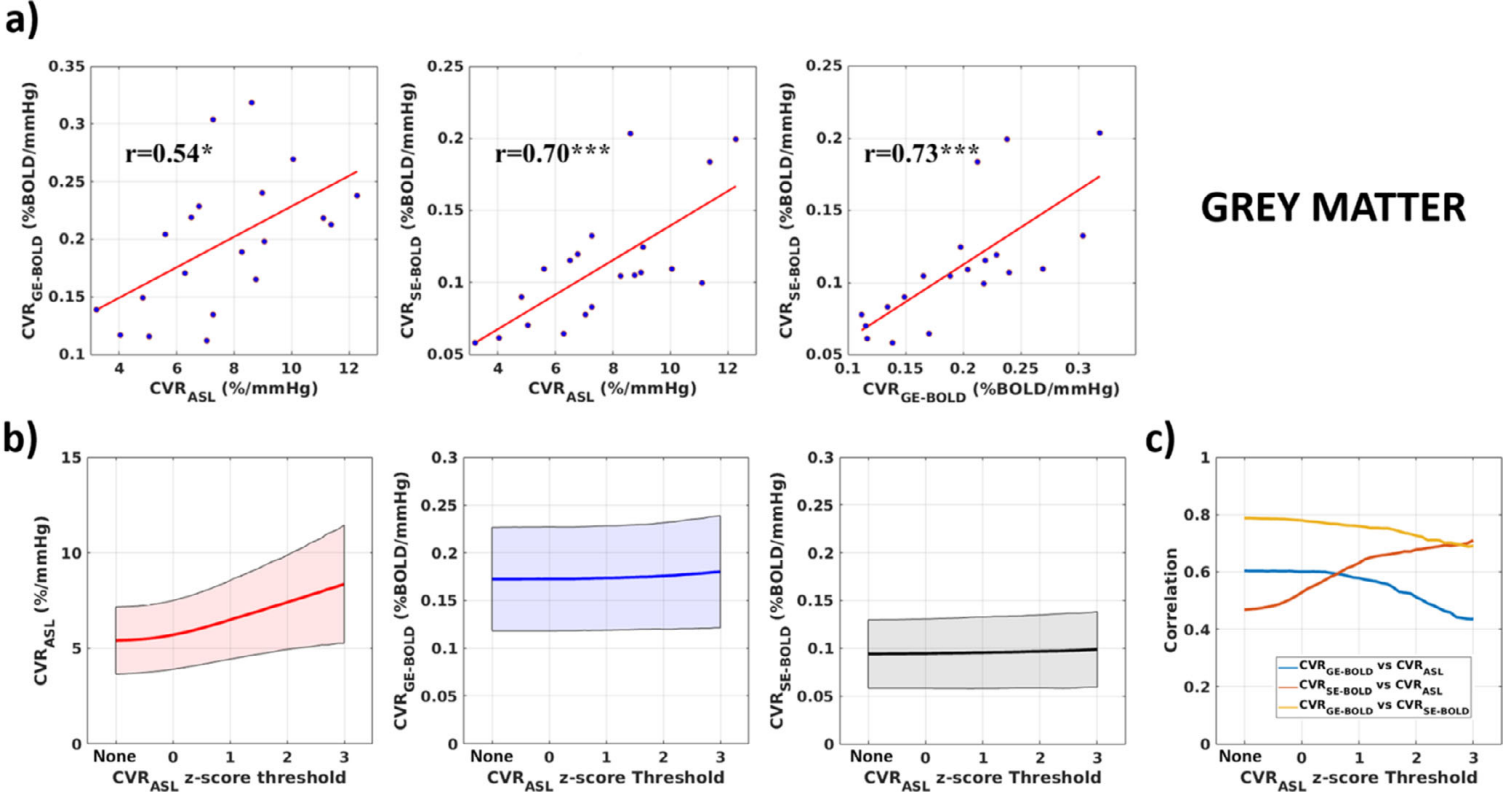
(a) Correlation plots across subjects for global CVR estimates in the gray matter (GM, voxel CVR_ASL_ *z*‐score > 2). (b) Subjects average (and standard deviation) values for the global CVR estimates as a function of the voxelwise statistical confidence (*z*‐score threshold) of CVR_ASL_. (c) Correlation values between the CVRs computed with the different fMRI approaches as a function of the voxelwise statistical confidence (*z*‐score threshold) of CVR_ASL_.

Figure 5a shows the across subjects correlation plots between the global CVR measures in GM when considering voxels with CVR_ASL_ *z*‐score > 2 (*p* < 0.05). Pearson's correlations were *r* = 0.54 (*p* < 0.05) for CVR_GE‐BOLD_ versus CVR_ASL_, *r* = 0.73 (*p* < 10^−3^) for CVR_SE‐BOLD_ versus CVR_ASL_, and *r* = 0.70 (*p* < 10^−3^) for CVR_SE‐BOLD_ versus CVR _GE‐BOLD_.

Figure 5b shows average (and standard deviation) values for CVR_ASL_, CVR_GE‐BOLD_, and CVR_SE‐BOLD_ in GM as a function of the CVR_ASL_ *z*‐score threshold employed. Without thresholding, CVR values in GM were CVR_ASL_ = 5.3%/mmHg ± 1.8%/mmHg (Coefficient of Variation, CoV, 34%), CVR_GE‐BOLD_ = 0.18% BOLD/mmHg ± 0.05% BOLD/mmHg (CoV, 28%), and CVR_SE‐BOLD_ = 0.09% BOLD/mmHg ± 0.03% BOLD/mmHg (CoV, 33%). Average subject‐wise and voxel‐wise *z*‐score values in the GM were CVR_ASL_ *z*‐score = 2.52 ± 0.66, CVR_GE‐BOLD_ *z*‐score = 6.38 ± 2.09, CVR_SE‐BOLD_ *z*‐score = 3.21 ± 0.72.

Average GM CVR_ASL_ estimate tended to increase at increasing values for the CVR_ASL_ *z*‐score threshold.

Figure 5c shows correlations between the three CVRs as a function of CVR_ASL_ *z*‐score threshold employed (Figure 5).

**FIGURE 6:**
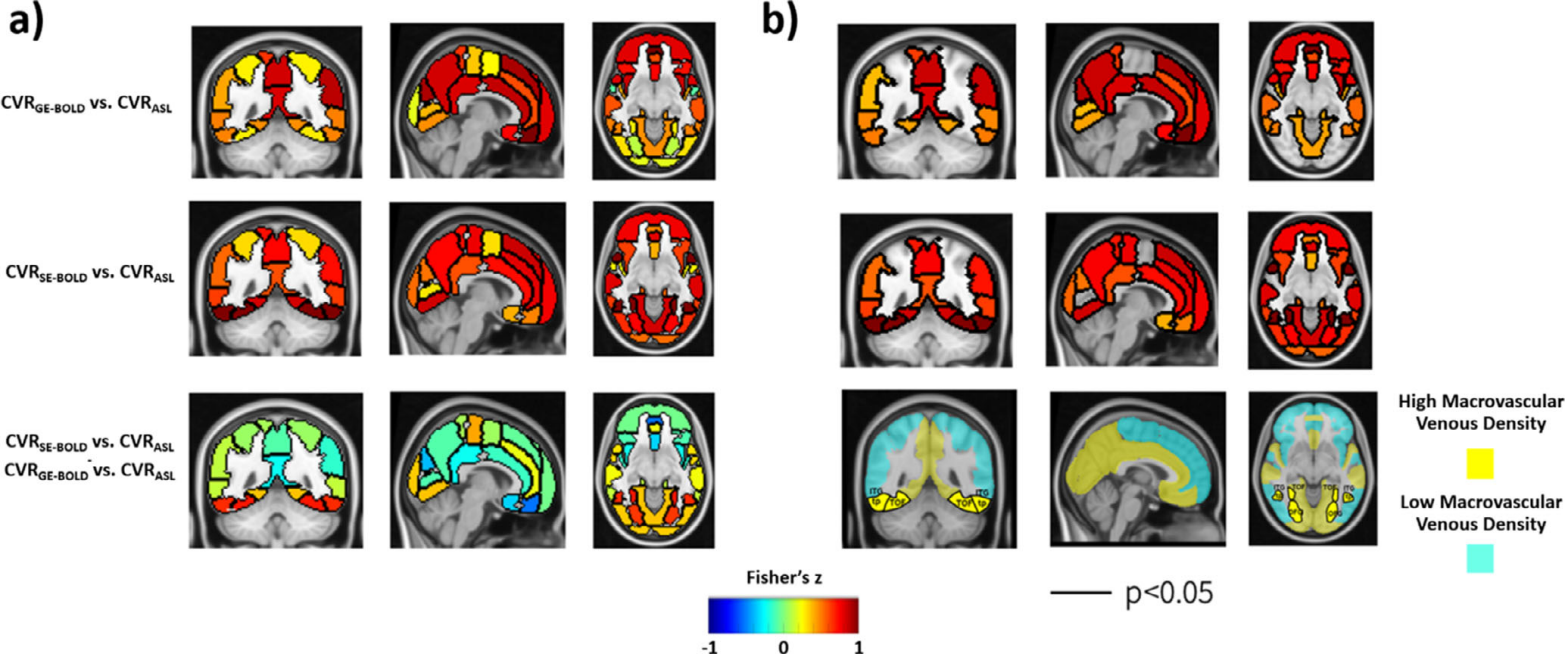
(a) Coronal, sagittal, and axial slices of the Fisher‐transformed correlation maps in MNI space computed across subjects within the ROIs of the Harvard Oxford Atlas (48 bilateral ROIs) for CVR_GE‐BOLD_ versus CVR_ASL_ (top row), CVR_SE‐BOLD_ versus CVR_ASL_ (middle row), and for their difference (CVR_SE‐BOLD_ vs. CVR_ASL_—CVR_GE‐BOLD_ vs. CVR_ASL_). (b) Same maps as in (a) but only with statistically significant ROIs (*p* < 0.05, FDR corrected). For the difference between Fisher‐transformed correlations (bottom row), significant regions are transparent and are only outlined in black, while the two‐color image represents a binarization of the Atlas based on previously reported macrovascular venous densities (high density vs. low density, above and below the 50th percentile, respectively) [28].

Figure 6a shows coronal, sagittal, and axial slices of the CVR_GE‐BOLD_ versus CVR_ASL_ and CVR_SE‐BOLD_ versus CVR_ASL_ Fisher Transformed correlation maps displayed in MNI space and computed across subjects for the parcellated ROIs (top and middle row, respectively). Also, the difference between the Fisher Transformed correlations of CVR_SE‐BOLD_ versus CVR_ASL_ and of CVR_GE‐BOLD_ versus CVR_ASL_ is reported (bottom row). Figure 6b shows the same maps but thresholded based on statistical significance (*p* < 0.05, FDR corrected). For the difference between correlations, significant regions are transparent and are only outlined in black. The two‐color image represents a binarization of the Atlas space based on macrovascular venous densities (high density vs. low density, above and below the 50th percentile, respectively) reported by Bernier and colleagues using a procedure based on SWI [28]. All three ROIs with significantly higher correlation of CVR_SE‐BOLD_ versus CVR_ASL_ compared to CVR_GE‐BOLD_ versus CVR_ASL_ (inferior temporal gyrus, temporooccipital part, ITGtp, occipital fusiform gyrus, OFG, temporal occipital fusiform cortex, TOF) fall into the group of high‐venous density ROIs.

**FIGURE 7:**
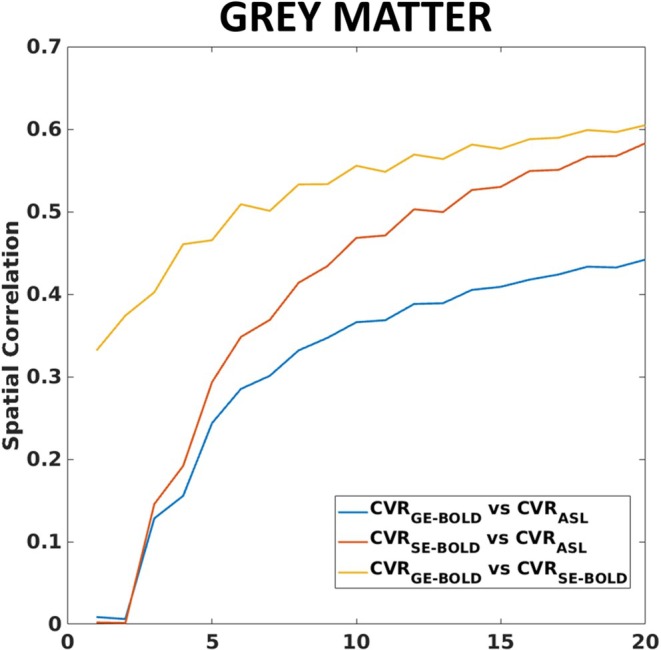
Spatial correlations in the gray matter (GM) between the three CVRs computed in the MNI space as a function of the number of subjects averaged (bootstrapping used on each subjects numerosity).

Correlations between CVRs within ROIs were primarily positive and were generally higher (and statistically more significant) for the CVR_SE‐BOLD_ versus CVR_ASL_ compared to CVR_GE‐BOLD_ versus CVR_ASL_ (Figure 7).

Figure 7 reports the spatial correlation function of the number of subjects averaged. A clear increase in correlation as more subjects were included in the averaging is visible. Pearson's correlation values when considering all subjects of the study were *r* = 0.43 for CVR_GE‐BOLD_ versus CVR_ASL_, *r* = 0.58 for CVR_SE‐BOLD_ versus CVR_ASL_, and *r* = 0.63 for CVR_SE‐BOLD_ versus CVR_GE‐BOLD_ (all *p*'s < 10^−3^).

## 4 Discussion

In this report, we present a study where GE‐BOLD and SE‐BOLD CVRs were compared to CVR estimated via ASL in the GM. All measurements were concurrently acquired using a newly developed fMRI sequence (Figure 1) during BH (Figures 2 and 3).

Despite the low SNR associated with functional ASL [48], particularly in the WM, the perfusion‐weighted ASL sequence is expected to quantify the physiological CVR, that is the percentage change in CBF per unit change in vasodilatory stimulus. In contrast, BOLD signals, while easier to implement and capable of delivering a higher SNR—especially for the GE version—produce semi‐quantitative measures of CVR not based on a single physiological quantity [14]. In fact, while BOLD is sensitive to changes in CBF during an isometabolic vasodilatory stimulus, it is also sensitive to the baseline dHb content [16]. Notably, the GE signal, with its well‐documented high sensitivity to veins [49, 50], can vary significantly as it probes both the microvascular and the macrovascular compartments [18]. Conversely, the refocusing pulse used in the SE‐BOLD method, despite reducing the expected BOLD signal modulation due to diffusion‐related effects, renders the T2‐weighted signal sensitive primarily to the microvasculature [21].

Qualitatively, the findings supported our initial hypothesis. CVR_GE‐BOLD_ and CVR_SE‐BOLD_ maps delivered expected characteristics, with the CVR_GE‐BOLD_ maps showing larger hyperintensities than the CVR_SE‐BOLD_ map in the presence of known large venous vessels (e.g., superior sagittal sinus, Figure 4a,b). Despite the lower signal change in response to BH of SE‐BOLD compared to GE‐BOLD (around 50% lower, Figure 4a,b), second‐level across‐subjects' *t*‐score maps were comparable between GE‐BOLD and SE‐BOLD CVRs, with *t*‐scores often above 10 in the GM and above 5 in the WM. This is suggestive of a similar sensitivity in detecting CVR for SE and GE when considering a group of participants. This effect could have been induced by the reduced influence of the variability in the volume of venous compartment in the SE signal compared to GE signal. Significantly lower *t*‐scores were obtained for CVR_ASL_, in particular in the WM (Figure 4c), highlighting the low SNR of ASL.

In the GM, average values identified for the CVRs were consistent with previous findings (Figure 5b) with average CVR_ASL_ in GM slightly above 5%/mmHg when not applying any statistical thresholds [24]. CVR_BOLD_ average values in the GM were around 0.17% BOLD/mmHg and 0.09% BOLD/mmHg for CVR_GE‐BOLD_ and CVR_SE‐BOLD_, respectively. As expected, confidence in the subject‐wise and voxel‐wise estimate was lowest for CVR_ASL_ (average *z*‐score around 2.5) and highest for CVR_GE‐BOLD_ (average *z*‐score around 6.5), with CVR_SE‐BOLD_ in‐between (average *z*‐score around 3.2).

Quantitative correlation analysis between the three CVRs revealed that both CVR_GE‐BOLD_ and CVR_SE‐BOLD_ were correlated across subjects in GM with the CVR_ASL_ (Figure 5a), with higher correlation for CVR_SE‐BOLD_. Moreover, the coefficient of variation (CoV) of CVR_SE‐BOLD_ was closer to CVR_ASL_ than was CVR_GE‐BOLD_.

Nonetheless, a portion of the variance in the GE‐BOLD and SE‐BOLD CVR was not explained by the physiological CVR. This discrepancy may be attributed to random noise variability or variability in other physiological parameters to which BOLD is sensitive, in particular CBV. Also, the higher correlation observed between CVR_ASL_ and CVR_SE‐BOLD_ compared to the correlation between CVR_ASL_ and CVR_GE‐BOLD_ may indicate a smaller contribution of veins in the variance of the SE signal compared to the GE signal. Importantly, these effects were evident when selecting ASL voxels with high confidence in the CVR estimate (CVR_ASL_ *z*‐score > 2, *p* < 0.05). While the GE‐BOLD weighting showed a decrease in correlation, the SE‐BOLD CVR correlation with the CVR_ASL_ was found to be higher for higher CVR_ASL_ *z*‐score thresholds (Figure 5c). At a *z*‐score threshold slightly above 0, the difference in the two correlations actually inverted the sign. Nonetheless, the larger correlation of CVR_SE‐BOLD_ with CVR_ASL_ compared to CVR_GE‐BOLD_ when only statistically significant CVR_ASL_ within each voxel were considered highlights the better performance of SE‐BOLD to probe the physiological CVR. It is important to note that while the *z*‐score thresholding approach increases the reliability of the CVR_ASL_ estimate, it can introduce an overestimation bias in CVR_ASL_ (Figure 5b) caused by the statistical approach [51]. However, this method does not statistically bias the estimation of correlations between the three CVRs, which are the key variables in our analysis. Nonetheless, the estimation of CVRs and their correlations may still be influenced by the spatial effects of the voxel selection method. The z‐score thresholding based on the significance of CVR_ASL_ may select voxels with more brain parenchyma and microvasculature while disregarding those with large veins. However, given that CVR_GE‐BOLD_ remains relatively constant across varying thresholds (Figure 5b), this effect appears negligible (at least at a global level), with the more significant impact stemming from the increased SNR of CVR_ASL_. Additionally, a minor residual effect from the voxel selection method that reduces the influence of veins should improve the between‐subject correlations of CVR_GE‐BOLD_ with CVR_ASL_ as a function of *z*‐score thresholding value, which goes against our findings, thus further supporting the results obtained.

These findings suggest the potential utility of BOLD weighting, particularly the SE, to probe CVR effectively.

The ROI analysis based on the Harvard–Oxford Atlas confirmed the global findings, with across‐subject correlations being overwhelmingly positive but with generally higher correlation values for CVR_ASL_ with CVR_SE‐BOLD_ compared to CVR_GE‐BOLD_ (Figure 6a). When looking at differences in correlations, CVR_SE‐BOLD_ was significantly more correlated to CVR_ASL_ than CVR_GE‐BOLD_ in only three ROIs (inferior temporal gyrus, temporooccipital part, ITGtp, occipital fusiform gyrus, OFG and temporal occipital fusiform cortex, TOF, *p* < 0.05, FDR corrected). Importantly, all three ROIs were in the group of regions exhibiting high macrovascular venous density (based on binarization at the 50th percentile), as measured from previous work based on SWI (Figure 6b) [28]. While other factors may contribute to the spatial variability in across‐subjects correlations between CVRs, such as susceptibility artifacts and cerebrospinal fluid partial volume contamination, this analysis suggests that the SE‐BOLD is less sensitive to macrovascular venous contamination than GE‐BOLD.

Considering instead the spatial comparison of CVR maps, we identified similar effects, with the CVR_SE‐BOLD_ exhibiting a higher spatial correlation with the CVR_ASL_ than CVR_GE‐BOLD_ (Figure 7). However, to highlight these effects, it was necessary to increase confidence in the CVR_ASL_ maps by averaging them across subjects. As the number of subjects increased, the absolute correlations and the difference in correlation between the CVR_GE‐BOLD_ and CVR_SE‐BOLD_ with the CVR_ASL_ also increased. On a single‐subject basis, the variability in the CVR_ASL_ maps likely has a significant contribution from noise. Since Hb in blood and venous saturation tend to be homogeneous across the space, at least in healthy subjects [17], these results highlight the likely confounding effect of veins in probing CVR with BOLD, particularly for the GE weighting.

Notably, single subject and average CVR images for the ASL clearly showed an unsuitability of the method to quantify CVR in the WM. The PLD used in this study (1.5 s) was rather short in comparison to the long bolus arrival times expected for WM. CVR_ASL_ appeared increased in the WM but with low confidence across subjects (low *t*‐score values, Figure 4c) plausibly due to the effects of a high noise level in the functional perfusion signal and of an interaction between incomplete bolus delivery and modulation in the transit time between normocapnia and hypercapnia in the WM.

Notably, in this study we acquired all three fMRI signals simultaneously and this approach should increase the reliability of the comparison between CVRs, as it eliminates the effects of variability in task performance, which is particularly relevant when using breath‐holding to induce hypercapnia. Moreover, it also eliminates effects of temporal fluctuations in brain physiology. However, the study has several limitations that should be acknowledged.

First, the CVR measured from ASL may be affected by transit‐time variability, partial volume effects, and changes in labeling efficiency due to increased blood velocity during hypercapnia [30, 31]. Second, the confidence in CVR maps may be diminished due to the slow sampling rate that was made necessary by the use of ASL [44]. The TR of 5 s, while sufficiently short to capture the signal response to BH without bias, may have reduced the CVR measurement precision relative to that which could have been estimated with a shorter TR. Third, the spatial resolution was limited due to the necessity of integrating the perfusion signal. Higher‐resolution imaging techniques may be implemented in future studies to reduce partial volume effects and provide more detailed spatial information regarding venous contamination in CVR estimates based on the GE and SE BOLD fMRI weighting. Fourth, BH increases movement‐related noise when compared to CO_2_ exogenous administration. The SE signal, which exhibits T2‐weighted hyperintensity in cerebrospinal fluid, may be more significantly affected by movement‐related noise when compared to GE. However, all images underwent movement correction, and the results do not suggest a strong contribution from movement‐related noise [46]. Fifth, hypercapnia has been reported to induce a mild modification in oxygen consumption [52, 53, 54]. A change in oxygen metabolism during hypercapnia would be an additional confound in the estimation of the CVR from the BOLD signal. Lastly, this study was conducted on healthy subjects, where the CVR presents limited variability [18]. Thus, it is recommended that the study be extended to include patients with cerebrovascular diseases, where a larger variability in CVR [14, 55], but also in other physiological parameters, may be observed. This extension would also enhance the understanding of altered CVR in many neurological conditions.

## 5 Conclusions

This fMRI study on healthy subjects demonstrates that both gradient‐echo and spin‐echo BOLD CVR correlate with ASL‐derived physiological CVR, particularly when sufficient confidence in the ASL modulation is achieved through the selection of statistically significant voxels or by averaging. The SE‐BOLD signal, despite having a lower signal‐to‐noise ratio compared to the GE‐BOLD signal, exhibits similar statistical power at the group level and stronger alignment with ASL, likely due to reduced contamination from venous macrovasculature in the CVR estimate compared to GE. The developed MRI sequence, which allowed for the concurrent acquisition of all three fMRI weightings, highlights the potential utility of BOLD recordings in effectively probing CVR. In particular, the SE weighting may be considered an alternative to functional ASL, which is limited by the low signal‐to‐noise ratio when trying to produce voxelwise single‐subject CVR maps.

Extending the study to include patients with cerebrovascular diseases could enhance the understanding of CVR variations and aid in the validation of fMRI methodologies in diverse clinical contexts. This could ultimately contribute to a deeper understanding of the role of altered CVR in neurological conditions.

## Funding

This work was supported by the NextGenerationEU, Italian Ministry of University and Research (MUR), Research National Program (PNR) and Projects of National Relevance (PRIN) (2022BERM2F, 2022MHMSSJ), the NextGenerationEU, Italian MUR, National Plan for Recovery and Resilience (PNRR), and PRIN (P20225AEEE, P2022ESHT4), the NextGenerationEU under the National Plan for Recovery and Resilience (PNRR), Italian MUR (ECS00000041‐VITALITY, PE0000006‐MNESYS). Emma Biondetti was supported by the European Union's Horizon Europe research and innovation programme under the Marie Skłodowska‐Curie (101066055‐HERMES). Alessandra Caporale was partially supported by the Fund for the Promotion and Development of Policies of the National Research Program, as per DM 737/2021 issued by the Italian MUR. Manuela Carriero was partially supported by Siemens Healthineers. Michael Germuska is supported by the Wellcome Trust (220575/Z/20/Z), and received funding from the Engineering and Physical Sciences Research Council (EP/S025901/1).

## Conflicts of Interest

Fabrizio Fasano and Domenico Zacà are employees of Siemens Healthineers, company producer of the scanner used to acquire the data presented in the study. The other authors declare no competing financial and non‐financial interests.

## Supporting information


**Figure S1:** Depiction of the processing involved in the extraction of the cerebrovascular reactivities (CVRs). The GE‐BOLD, SE‐BOLD, and ASL volumes were motion‐corrected, registered to the proton density image (M0), distortion‐corrected and filtered. Structural MRIs were segmented and registered to the M0 image. PetCO_2_ traces were extracted from the expired CO_2_ signals and filtered. Voxelwise signals were regressed against the PetCO_2_ trace with a time lag allowed of ±10 s (two samples) to extract the CVRs.

## Data Availability

The data that support the findings of this study are available from the corresponding author upon reasonable request. The code to compute CVR maps is available at: https://github.com/chiarell/Hypercapnic‐Calibrated‐fMRI.
