# Supplementary material for: FMRI Approaches to Mapping Cerebrovascular Reactivity: Comparison of Gradient Echo BOLD and Spin Echo BOLD With Arterial Spin Labeling
Source: Magn Reson Med. 2026 Feb 22;95(6):3309–21. doi: 10.1002/mrm.70305 (PMC13049236; doi:10.1002/mrm.70305)
Supplement: Supplementary file 1 — Figure S1: Depiction of the processing involved in the extraction of the cerebrovascular reactivities (CVRs). The GE‐BOLD, SE‐BOLD, and ASL volumes were motion‐corrected, registered to the proton density image (M0), distortion‐corrected and filtered. Structural MRIs were segmented and registered to the M0 image. PetCO2 traces were extracted from the expired CO2 signals and filtered. Voxelwise signals were regressed against the PetCO2 trace with a time lag allowed of ±10 s (two samples) to extract the CVRs. [file MRM-95-3309-s001.docx]

**
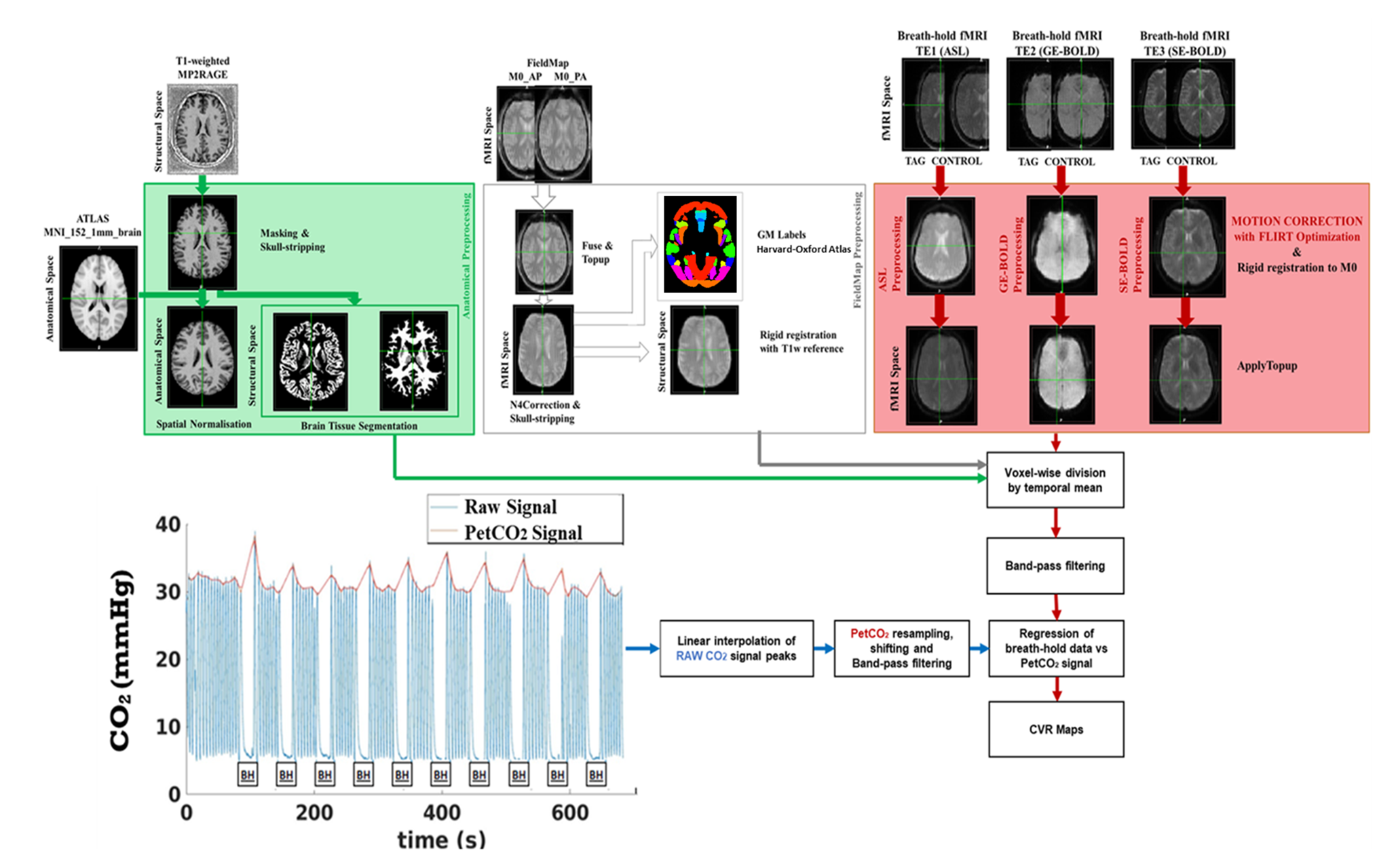
**

**Figure S1.** Depiction of the processing involved in the extraction of the cerebrovascular reactivities (CVRs). The GE-BOLD, SE-BOLD, and ASL volumes were motion-corrected, registered to the proton density image (M0), distortion-corrected and filtered. Structural MRIs were segmented and registered to the M0 image. PetCO_2_ traces were extracted from the expired CO_2_ signals and filtered. Voxelwise signals were regressed against the PetCO_2_ trace with a time lag allowed of ±10 seconds (2 samples) to extract the CVRs.
